# The Use of Single Drop Microextraction and Field Amplified Sample Injection for CZE Determination of Homocysteine Thiolactone in Urine

**DOI:** 10.3390/molecules26185687

**Published:** 2021-09-20

**Authors:** Krystian Purgat, Izabella Kośka, Paweł Kubalczyk

**Affiliations:** 1 Department of Environmental Chemistry, Faculty of Chemistry, University of Lodz, 163 Pomorska Str., 90-236 Lodz, Poland; izabella.koska@edu.uni.lodz.pl; 2 Doctoral School of Exact and Natural Sciences, University of Lodz, 12/16 Banacha Str., 90-237 Lodz, Poland

**Keywords:** capillary electrophoresis, field amplified sample injection, homocysteine thiolactone, off-line single drop microextraction, urine

## Abstract

Two cheap, simple and reproducible methods for the electrophoretic determination of homocysteine thiolactone (HTL) in human urine have been developed and validated. The first method utilizes off-line single drop microextraction (SDME), whereas the second one uses off-line SDME in combination with field amplified sample injection (FASI). The off-line SDME protocol consists of the following steps: urine dilution with 0.2 mol/L, pH 8.2 phosphate buffer (1:2, *v/v*), chloroform addition, drop formation and extraction of HTL. The pre-concentration of HTL inside a separation capillary was performed by FASI. For sample separation, the 0.1 mol/L pH 4.75 phosphate buffer served as the background electrolyte, and HTL was detected at 240 nm. A standard fused-silica capillary (effective length 55.5 cm, 75 μm id) and a separation voltage of 21 kV (~99 μA) were used. Electrophoretic separation was completed within 7 min, whereas the LOD and LOQ for HTL were 0.04 and 0.1 μmol/L urine, respectively. The calibration curve in urine was linear in the range of 0.1–0.5 μmol/L, with R^2^ = 0.9991. The relative standard deviation of the points of the calibration curve varied from 2.4% to 14.9%. The intra- and inter-day precision and recovery were 6.4–10.2% (average 6.0% and 6.7%) and 94.9–102.7% (average 99.7% and 99.5%), respectively. The analytical procedure was successfully applied to the analysis of spiked urine samples obtained from apparently healthy volunteers.

## 1. Introduction

The only source of homocysteine (Hcy) in the human organism is methionine (Met) delivered with food, especially in diets rich in meat. Normally, Hcy is converted to cysteine or back to Met through two different metabolic pathways called transsulfuration and remethylation, respectively. However, due to some genetic or nutritional deficiencies, Hcy metabolism is disturbed as a result of Hcy accumulation [1,2]. An increased total Hcy content in plasma, called hyperhomocysteinemia [3], is connected to cardiovascular [4,5] and neurodegenerative disorders [6]. Hcy can undergo conversion to Hcy-thiolactone (HTL) throughout error-editing reactions by methionyl-tRNA synthetase instead of Met [7]. Many studies performed on animals and cell cultures have proven that HTL is very cytotoxic [8]. Moreover, the previous study showed that HTL levels are elevated in hyperhomocysteinemic mice [9]. The harmful influence of HTL on the organism comes from two major HTL reactions in serum: (1) protein *n*-homocysteinylation and (2) enzymatic hydrolysis to Hcy, followed by protein S-homocysteinylation [1]. HTL reacts with protein to form an Hcy-containing adduct, in which the carboxyl group of Hcy is linked by an amide bond to the ε-amino group of a protein lysine residue [10,11,12]. Due to this modification, HTL changes proteins’ structure [12,13,14], decreases the physiological activity of proteins [12,13,15], and has toxic effect on cells as well [16]. Since under physiological conditions HTL exists in the form of a neutral base, it can freely diffuse through cell membranes [1]. As a harmful metabolite of Hcy, most of the HTL produced in tissues is eliminated via urine [9,17], because the metabolic excretion of HTL is typical of waste or toxic products in humans [1]. Consequently, the concentrations of HTL in urine are approximately 100-fold higher than those in plasma [17,18]. Therefore, urine is a bodily fluid commonly utilized for analysis because it is easy to obtain from patients.

Due to the fast development of chemical industries and the poor condition of the environment, attention is paid to respecting green chemistry recommendations and rules that should be implemented as part of the practice. Therefore, analytical laboratories direct their work towards minimizing harmful effects on the environment and people by eliminating or reducing toxic chemicals and reagents from analytical procedures [19].

One of the main drawbacks of CE is its low concentration sensitivity, particularly in comparison to HPLC. In order to lower the limit of quantification (LOQ) of a method, some techniques, such as sample stacking inside a CE system, or extraction, are utilized. Liquid-liquid extraction is commonly used for sample preparation or separation, as well as the preconcentration of analytes. Unfortunately, liquid-liquid extraction also exhibits significant disadvantages, i.e., it uses a large volume of potentially toxic organic solvents, it is tedious, and it is time-consuming [20,21]. Therefore, cheap, easy to use, and environmentally friendly procedures based on liquid-phase microextraction have been developed [22]. One of the liquid phase microextraction techniques is single-drop microextraction (SDME). This microextraction technique was first used in the middle of the 1990′s [23], and it is a powerful tool that can be realized either outside (off-line) or inside (on-line) a GC, HPLC, and CE system. One of the most important advantages of SDME in the context of environmental protection concerns the significant reduction in the consumed toxic organic solvents. The next favorable feature of SDME is its capacity for sample purification. Moreover, SDME is characterized by a high enrichment factor that, in combination with its simplicity and inexpensive implementation in analytical procedures, makes SDME a very attractive technique for analyte preconcentration [24].

Nowadays, different methods that use GC [8,25,26], HPLC [17,18,27,28,29,30], and CE [3,31,32] for the quantification of HTL in biological samples are known. Almost all of these methods, except procedures based on mass spectrometry and direct UV detection [3,25,26,27,28], depend on the pre-, on- or post-column derivatization of HTL. Furthermore, some methods use eluents containing a mixture of harmful organic solvents—mostly acetonitrile and buffer components or ion-pairing reagents. Unfortunately, these compounds have corrosive or cytotoxic properties, and are very stable in the environment. In the case of CE, the limit of detection (LOD) based on concentration is insufficient for many practical applications. Therefore, several on-line or on-capillary methods have been developed to pre-concentrate analytes inside the capillary before separation and detection. Field amplified sample injection (FASI) is a stacking procedure in which the sample is electrokinetically injected into the capillary. The sample is usually prepared in a buffer solution characterized by much lower conductivity than BGE. Analyte ions enter the capillary as a result of electrophoresis and electroosmosis. Due to differences in electric field strength between the sample and BGE, when they reach the BGE zone, the accelerated analytes suddenly undergo a decrease in migration velocities, and stacking. Additionally, depending on the applied polarization, cations or anions are injected into the capillary, and thus the sample matrix is simplified [33]. After the FASI is finished, the stacked analytes enter BGE and separation by CZE proceeds.

In this paper, two CE methodologies that use single-drop microextraction (SDME) and field amplified sample injection (FASI) were developed. These procedures were compared with each other in order to choose the most efficient one for the simple, sensitive and accurate determination of HTL in urine. The first methodology (method A) uses only the SDME technique in off-line mode. The second procedure (method B) utilizes two combined preconcentration techniques, i.e., SDME in the off-line mode together with on-capillary FASI.

## 2. Results and Discussion

### 2.1. Capillary Zone Electrophoresis

Several parameters, such as the concentration and pH of BGE, separation voltage, as well as capillary temperature for CZE analysis, were optimized (all data are included in Supplementary Data). The first checked parameter was the concentration of BGE, in the range of 0.05–0.15 mol/L. According to Figure S1 (in the Supplementary Material), the largest signal was obtained for 0.1 mol/L phosphate buffer. The pH of the separation buffer in the range of 3.0 to 6.0 was tested as well. The best results were obtained for a pH value of 4.75. The influence of the applied voltage on sample separation was also studied by plotting the relationship between current and voltage. Based on the prepared plot (Figure S2 in Supplementary Material), the most efficient separation was achieved with the voltage of 21 kV. The capillary temperature ranging from 21 to 26 °C was the last parameter tested. It was found that with a rise in the temperature, the peak area increased. The peak height also increased, but only up to the temperature of 23 °C. Since further increases in the temperature caused reductions in peak height, 23 °C was chosen as the capillary temperature. The average migration time of HTL in urine under optimized conditions was 6.9 min, while the reproducibility of migration time was very good, with RSD value less than 2.1% (n = 10).

### 2.2. Field Amplified Sample Injection

In order to obtain the higher stacking efficiency in method B, parameters such as concentration of BGE, injection time, injection voltage, type and concentration of acid solution for sample preparation after SDME procedure, as well as the length of the water plug injected before and after the sample zone, were investigated in detail. The concentrations of Na_2_HPO_4_ and H_3_PO_4_ used for BGE composition were tested in the ranges of 0.05–0.10 mol/L and 0.5–2.0 mol/L, respectively. The pH of all BGEs was fixed at 4.75. The best results were obtained for BGE consisting of 0.06 mol/L Na_2_HPO_4_ and 1.5 mol/L H_3_PO_4_. The time and voltage of sample injection were studied in the range of 10 to 60 s and from 2 to 6 kV, respectively. It is well known that as the voltage and time increase during sample injection, the volume of the sample introduced into the capillary increases. Unfortunately, introducing too much sample into the capillary causes the widening of the peaks and reductions in resolution. In our procedure, we observed no increase in peak height with simultaneous peak broadening for times longer than 30 s. On the other hand, increasing the voltage to above 5 kV resulted in only a slight increase in peak height and peak area, with a simultaneous decrease in repeatability. Finally, we concluded that using a voltage of 5 kV for 30 s is optimal. Several acid solutions including H_3_PO_4_, HCOOH, CH_3_COOH, HCl and H_3_BO_3_ were tested to dissolve the extracted components of the sample. It was found that the use of boric acid yields the highest HTL signals. After the optimization of the concentration of this acid solution, the highest stacking efficiency was obtained for 0.0004 mol/L H_3_BO_3_. The influence of the water plug length introduced into the capillary before, after or before and after sample the zone was also studied. The best stacking effect was achieved with the hydrodynamic introduction of water plugs before and after the sample zone by applying a pressure of 10 mbar for 10 s and 10 mbar for 5 s, respectively.

### 2.3. Optimization of LLL-SDME Conditions

During the method development, three-phase SDME in off-line mode for sample clean up and HTL preconcentration was used. However, the liquid-liquid SDME was also tested. The extraction efficiency obtained was much better with the three-phase microextraction technique. The use of the third phase in the extraction procedure resulted in higher signal parameters, i.e., peak height and peak area. Several important parameters, such as sample pH, the concentration of the acceptor phase, organic solvent volume, and the time of two-step extraction (first extraction step from sample to organic layer and second one from organic layer to acceptor phase drop), where thoroughly optimized.

#### 2.3.1. Selection of Donor Phase pH

It is commonly known that if non-polar organic solvents are used for extraction, the proper selection of sample pH plays a key role in the effectiveness of the extraction. Molecules with no charge transfer more efficiently from the aqueous to the organic phase than molecules in the form of an ion. Since HTL possesses amino groups and the pKa for this compound is 6.67, the pH of a sample should be slightly alkaline. Therefore, the pH of the phosphate buffer for sample preparation was studied in the range from 5.5 to 8.2. For a pH of the buffer lower than 6.67, the extraction efficiency was very low, whereas for a higher buffer pH, the efficiency constantly increased up to pH 8.0. Although for pH values higher than 8.0 HTL the signal did not increase, a better repeatability of peak height and area was achieved at pH 8.2. For this reason, 0.2 mol/L of phosphate buffer, pH 8.2, was chosen for sample preparation.

#### 2.3.2. Selection of Organic Solvent Volume

**Figure 1:**
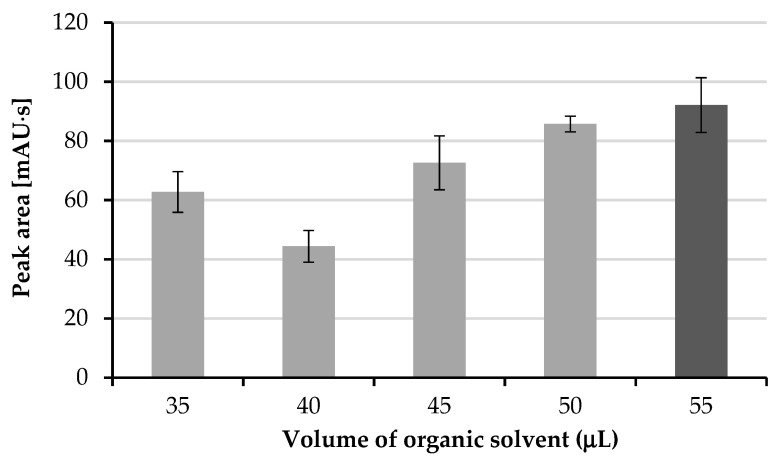
Influence of organic phase volume on peak area.

The organic solvent in LLL-SDME is a medium that separates the water solution of the donor and acceptor phases, as well as allowing for the permeation of an analyte from a sample to a drop. This organic layer is placed on the sample surface in order to enable the efficient passing of HTL from the donor sample to the acceptor drop. First, some solvents, such as dichloromethane, chloroform, toluene, ethyl acetate and methanol, were tested as organic solvent layers. Then, optimization of the volume of the organic layer was also performed. The best results were obtained for dichloromethane. Unfortunately, a dichloromethane layer tends to fall down on the bottom of the flask. On the other hand, the chloroform layer had better stability, but the area of its HTL peak was a bit smaller. Next, optimization of the volume of the chloroform layer was performed. According to the theoretical considerations [22,24], in liquid-liquid-liquid SDME, the thinner the organic layer, the higher the extraction efficiency. For this reason, an increased volume of chloroform was placed on the sample surface, starting with 35 μL. According to Figure 1, the peak area of HTL increased with the increase in chloroform volume. Unfortunately, for volumes higher than 55 μL, we had a problem with the organic layer falling to the bottom of the flask. On the other hand, when the volume of this layer was smaller than 50 μL, acceptor drop formation became difficult to operate. Hence a volume of the organic layer equal to 55 μL was used for further analysis.

#### 2.3.3. Selection of Acceptor Phase Concentration

**Figure 2:**
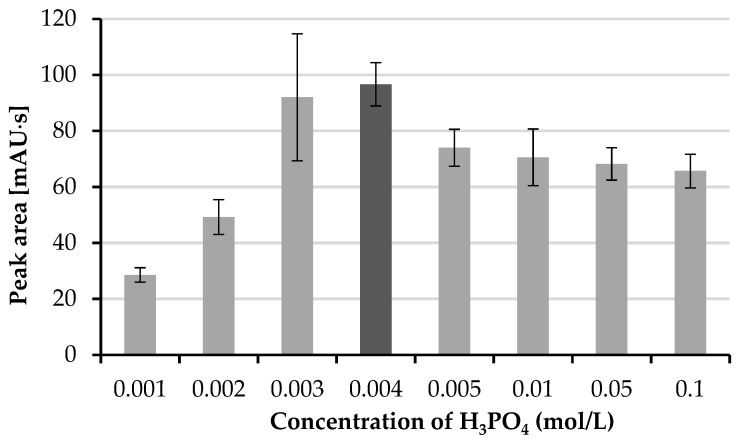
Influence of phosphoric acid concentration on peak area.

Due to the pH of the drop and the pKa of HTL, analyte molecules are captive in the drop of the aqueous acceptor phase. If the pH of the acceptor drop is below the pKa of HTL, its molecules are charged and do not permeate back to the organic phase, and then to the sample. Various types of acids were tested for use as the acceptor phase. The influence of the concentration of phosphoric acid over the extraction efficiency was tested in the range from 0.001 mol/L to 0.1 mol/L. The best results were received for 0.004 mol/L phosphoric acid (Figure 2).

#### 2.3.4. Selection of First Step Extraction Time

**Figure 3:**
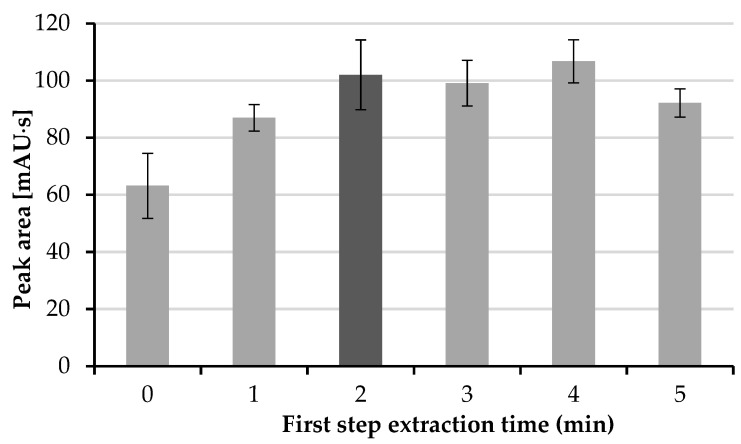
Influence of first step extraction time on repeatability and area of the peak.

The first step of extraction, i.e., from the donor phase to the organic phase, plays an important role in the extraction process. Hence, the time of this extraction step was tested in the range from 0 to 5 min, with 1 min intervals. It can be clearly seen (Figure 3) that the time of this step should last at least 2 min.

#### 2.3.5. Selection of Second Step Extraction Time

**Figure 4:**
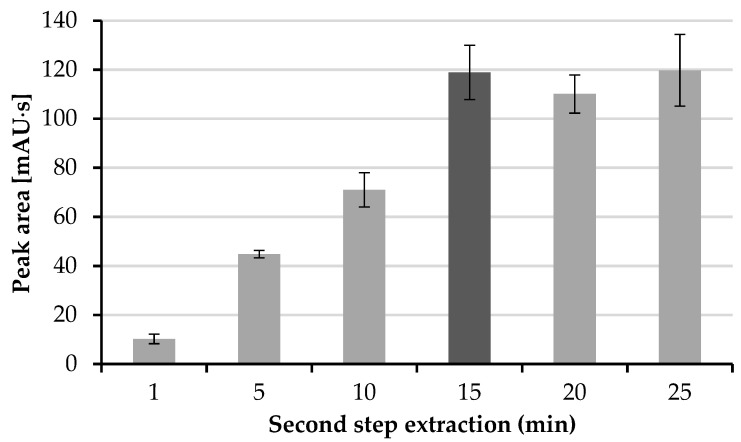
Influence of second step extraction time on peak area.

The second step of extraction is when the HTL molecule permeates from the organic layer into the drop of the acceptor phase. The time of this extraction step was investigated in the range from 1 to 25 min, with 5 min intervals. As can be expected, the elongation of the extraction time increases the efficiency of this process. According to Figure 4, the maximal efficiency is obtained after 15 min, whereas for longer extraction times, no increase in the peak area of HTL was observed. Therefore, a time of the second extraction step of 15 min was chosen.

### 2.4. Evaluation of Sensitivity Enhancement Factor

**Figure 5:**
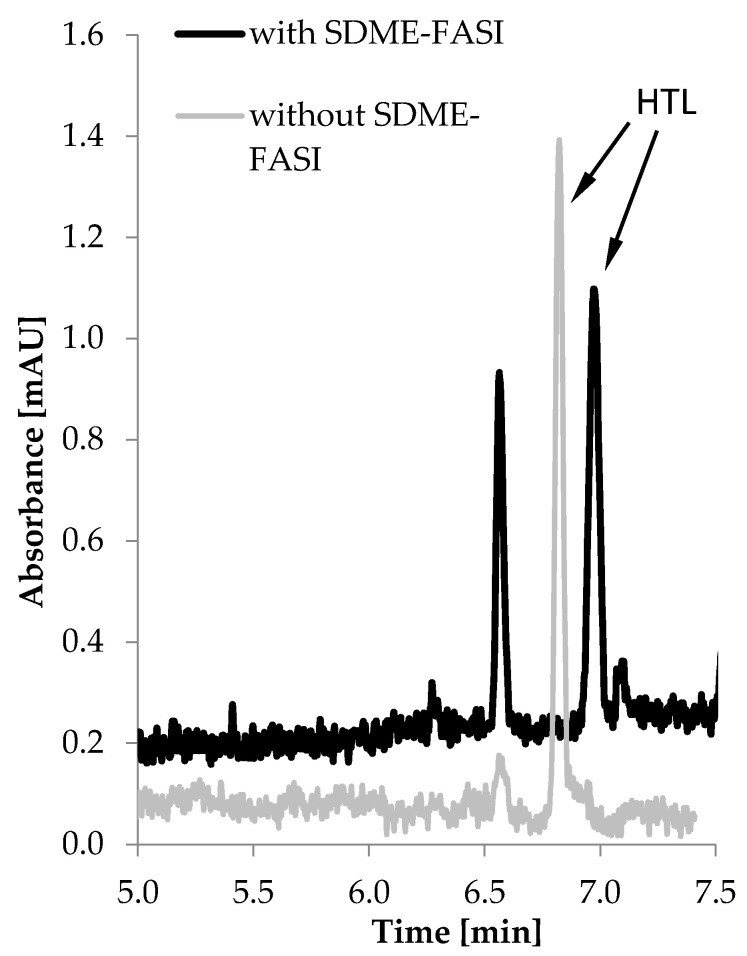
Representative electropherograms of human urine obtained with the use of the SDME-FASI-CZE method (black line) and a simple CZE method that does not utilize any concentration technique (gray line). The urine sample was spiked with known amounts of HTL equal to 0.4 μmol/L urine (for SDME-FASI-CZE) and 80 μmol/L urine (for CZE).

One of the most popular ways to establish the efficiency of an analyte preconcentration in a sample is the sensitivity enhancement factor (SEF). The SEF is calculated by the comparison of the peak height or peak area obtained in the method with a concentration step to that derived from a procedure without a concentration step. In order to calculate the SEF, the following equation was used:

(1) $${\mathrm{SEF}}_{A}=\frac{A\prime }{A}\times \frac{C}{C\prime }$$

where A′—peak area for urine analysis after concentration step, A—peak area for urine analysis without concentration step, C′—concentration of analyte in the sample for analysis with the use of concentration step, C—concentration of analyte in the sample for analysis without concentration step. This study was performed for method A and method B by preparing two urine samples in triplicate. The first sample was prepared according to the procedure described in Section 3.6 and analyzed with the use of concentration step. In method A, only the off-line SDME procedure was applied, whereas in method B on-line sample stacking (FASI), realized in the CE system after off-line SDME, was utilized. The second urine sample, not subjected to concentration, was introduced into the capillary by standard hydrodynamic injection (sample volume equal to about 2% of total capillary volume). The calculated SEF_A_ values for method A and method B amounted to 100 and 212, respectively. The representative electropherograms obtained after the analysis of the urine sample via method B and a protocol with no concentration step are shown in Figure 5.

### 2.5. Calibration and Validation Data

The elaborated methods were calibrated and validated in accordance with the criteria for biological samples analysis [34]. In the first step, the LOD and LOQ of the method were evaluated using the signal-to-noise ratio method, where the S/N ratios for LOD and LOQ were 3 and 9, respectively. The LOD and LOQ for HTL in urine were 0.12 μmol/L and 0.22 μmol/L via method A, and 0.04 μmol/L and 0.1 μmol/L via method B, respectively. The LOQs of the presented methods are similar to those of the MEKC-FASI (0.1 μmol/L urine) [3] and LC-MS/MS (0.5 μmol/L plasma) [27] methods. However, they are higher than those derived by the HPLC-FLD (0.02 μmol/L urine) [18], GC-MS (0.01 μmol/L urine 0.05 μmol/L saliva, and 0.0052 μmol/L plasma) [8,25,26] and HPLC-FLD (0.00036 μmol/L plasma) [30] protocols. The calibration of the proposed methods was achieved by plotting five-point calibration curves for HTL in urine in the concentration range from 0.22 to 0.50 μmol/L in triplicate for method A, and from 0.10 to 0.50 μmol/L in triplicate for method B. The calibration curves show a linear character in the tested concentration ranges, with correlation coefficients R^2^ = 0.9993 for method A and R^2^ = 0.9991 for method B. The relative standard deviation of the points of the calibration curve in urine varied from 1.6% to 13.8% (method A) and from 2.4% to 14.9% (method B). The recovery values for methods A and method B were within the ranges 99.8–101.1% and 95.7–102.6%, respectively. The intra- and inter-day precision and accuracy of the method were also evaluated. For this purpose, three concentrations representing the whole calibration range were tested, i.e., the first near the lower end, the second near the middle, and the third at the upper end of the standard curve. Both procedures are characterized by satisfactory precision values of less than 9% for method A and 11% for method B, as well as good accuracy values in the range from 93% to 100% and from 95% to 103%, respectively. The method precision, accuracy, and all other validation parameters comply with the criteria for biological sample analysis [34]. Taking into account the above, we are convinced that our method could be applied for the determination of HTL in human urine. All validation data are included in Table 1 and Table 2.

The sample preparation procedure of the above-described methods is simple and not time-consuming. The overall analysis times for method A (~30 min) and for method B (~40 min) were shorter than those of the previously reported procedures for MEKC-FASI in urine (~42 min) [3], HPLC-FLD in urine (~96 min) [18], GC-MS in urine (~120 min) [25], GC-MS in saliva (~60 min) [26], and GC-MS in plasma (~55 min) [8], while they were similar to those reported for HPLC-FLD in plasma (~24 min) [30] and SDME-CZE in urine (~21 min) [32], and longer than those reported for LC-MS/MS in plasma (~13.6 min) [27].

### 2.6. Determination of HTL in Human Urine

**Figure 6:**
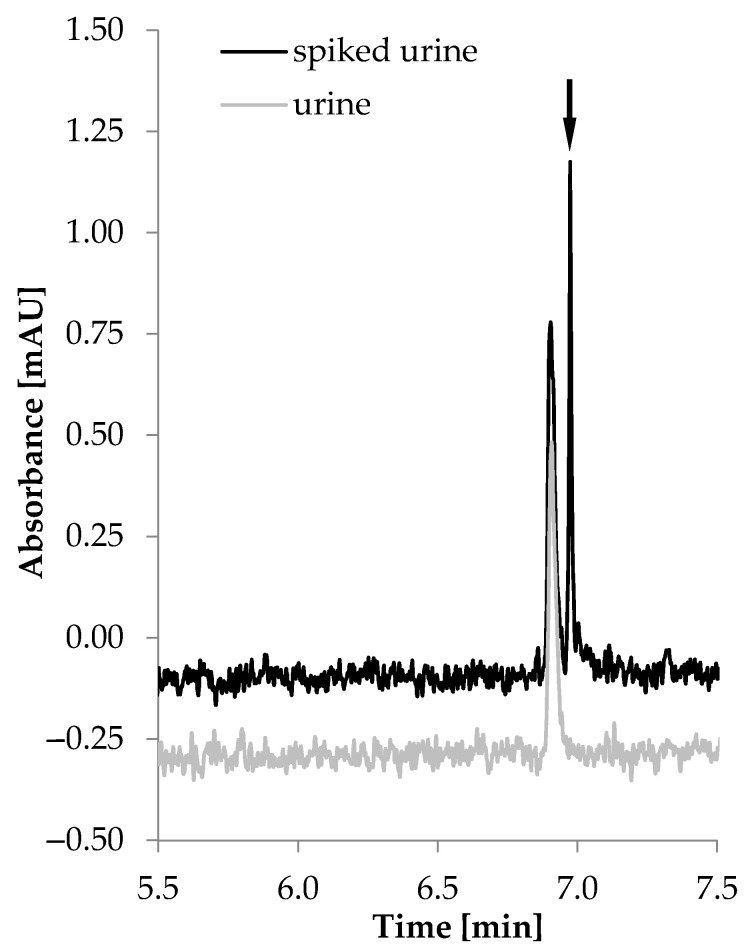
Electropherograms of blank urine sample (grey line) and urine sample spiked with 0.1 μmol/L HTL (black line).

After the optimization, calibration and validation of both analytical procedures, method B was chosen for HTL quantification in urine, given that it provided the highest SEF factor and the most satisfactory LOQ value. The representative electropherogram of a urine sample after off-line SDME and on-line FASI is shown in Figure 6.

Urine samples donated from 11 apparently healthy volunteers were prepared as described in Section 3.6, spiked with 0.1 μmol/L HTL, and subjected to SDME. After the extraction step, each urine sample was analyzed in accordance with method B, i.e., injected into a CE capillary by FASI and separated by CZE. The physiological human urine pH depends mainly on the diet, and ranges from 6 to 8. We did not observe the influence of urine pH on the obtained results. Since the microextraction process is quite selective and the applied electrokinetic sample injection promotes the introduction of cations and sample matrix simplification, we did not find any potential interferents. The HTL concentrations in the spiked urine samples taken from volunteers varied from 0.09 to 0.16 μmol/L urine. The obtained values were similar to the concentrations resulting from spiking, and the slight differences were caused by different contents of endogenous HTL. In turn, such a low concentration of endogenic HTL in urine is not surprising in samples from healthy people [3,17,18]. All data are included in Table 3.

## 3. Materials and Methods

### 3.1. Apparatus

All separation experiments were carried out using a computerized Agilent 7100 Capillary Electrophoresis System (Agilent Technologies, Waldbronn, Germany) equipped with a UV-Vis absorbance diode array detector and automatic injector. Bare fused-silica capillaries (Polymicro Technologies, Phoenix, AZ, USA) of different inner diameters and effective lengths were used during method development and sample separation. For the single-drop microextraction of HTL, a 50 μL Hamilton microsyringe (Hamilton, Reno, NV, USA) and SCHOTT TM 125 magnetic stirrer (SCHOTT, Mainz, Germany) was used. The identification of the HTL signal obtained during CE analysis was performed by the comparison of migration time and diode array spectra, taken at the time of analysis, with a corresponding set of data obtained by analyzing authentic compounds. All signal parameters, such as peak height, corrected peak area and migration times, were measured using Agilent ChemStation software. For the adjustment of buffer pH, a HANNA Instruments HI 221 pH-meter (HANNA Instruments, Cluj-Napoca, Romania) was used. All solutions needed during the experiments were prepared with the use of water purified by a MILLIPORE Milli-Q PLUS deionization System (MILLI-PORE, Watford, UK).

### 3.2. Chemical and Reagents

Sodium hydroxide (NaOH), sodium hydrogen phosphate (Na_2_HPO_4_), toluene (C_6_H_5_CH_3_) and chloroform (CHCl_3_) were from POCH (POCH, Gliwice, Poland). Phosphoric acid (H_3_PO_4_), dichloromethane (CH_2_Cl_2_) and methanol (CH_3_OH) were purchased from J.T. Baker (J.T. Baker, Deventer, Netherlands). Octadecyltrimethoxysilane (ODTS), ethyl acetate (CH_3_COOCH_3_) and d,l-Homocysteine thiolactone hydrochloride were received from Sigma (Sigma, St. Louis, MO, USA). To prepare stock standard solution of HTL (final concentration 0.1 mol/L) an appropriate amount of this compound was dissolved in water. All buffers needed for CE analysis were filtered through 0.2 μm pore size membranes. The pH values of the buffers used for CZE separation and sample preparation were adjusted by potentiometric titrations. The pH-meter was calibrated with standard pH solutions.

### 3.3. Capillary Preconditioning

Before first use, each capillary was preconditioned for 20 min with 1 mol/L NaOH solution, and then with 0.1 mol/L NaOH solution for 20 min, with deionized water for 2 min, and finally with background electrolyte (BGE) for at least 30 min. Each day before the CE analyses, the capillary was flushed with a 0.1 mol/L NaOH solution for 10 min, then with deionized water for 2 min, and then with BGE for 20 min in order to achieve a state of equilibration, whereas between runs the capillary was washed with BGE for 6 min in the case of method A and 10 min in the case of method B. At the end of the day, the capillary was flushed with deionized water and allowed to sit overnight.

### 3.4. Electrophoretic Conditions

The measurement experiments performed via method A were carried out with the use of 0.1 mol/L phosphate buffer (pH 4.75), which served as the BGE. In this case, 0.1 mol/L phosphoric acid was titrated with 0.1 mol/L sodium hydrogen phosphate solution. Urine components extracted from the sample were hydrodynamically introduced into the capillary. During CE separation, a voltage of 21 kV (~99 μA) was applied. The separation capillary (total length of 64 cm, effective length of 55.5 cm and 75 μm inner diameter) was kept at 23 °C. In turn, the electrophoretic conditions of method B were as follows: the BGE was an aqueous solution of 0.06 mol/L Na_2_HPO_4_, adjusted to pH 4.75 with 1.5 mol/L H_3_PO_4_; sample injection by FASI protocol. CZE analysis was performed at 21 kV, whereas the capillary (total length of 60 cm, effective length of 51.5 cm and 75 μm inner diameter) temperature was also set to 23 °C. During all electrophoretic analyses, the peaks were recorded at the analytical wavelength of 240 nm.

### 3.5. Human Urine Collection

For the urinary determination of HTL, the first morning urine samples were collected from apparently healthy volunteers (11 persons, 3 men and 8 women). These samples were either prepared immediately after collection or stored at −20 °C until analysis. Written informed consent forms were obtained from all volunteers, and this study was approved by the Bioethics Committee of the University of Lodz (15/KBBN-UL/III/2018).

### 3.6. Urine Sample Preparation

A 1.667 mL sample of urine was transferred into 5 mL calibrated flask, spiked with a known amount of HTL standard solution, made up to the volume by the addition of 0.2 mol/L phosphate buffer (pH 8.2), and thoroughly mixed. Next, a magnetic stirrer was put into the flask, and then, in order to start the extraction procedure, 55 μL of organic phase (chloroform) was cautiously placed on the top of the donor phase to form the thin meniscus of the organic layer on the sample’s surface.

### 3.7. SDME Procedure

Prior to the liquid-liquid-liquid microextraction (LLL-SDME) procedure, a microsyringe was appropriately prepared. First, the microsyringe was rinsed several times with methanol. Then, in order to improve the stability of the drop, the needle tip was immersed for 3 s in a surface coating solution (5% ODTS and 0.1% acetic acid in ethanol; after preparation of the mixture, condensation reaction allowed to proceed overnight) and then put aside. After 2 min, the microsyringe was rinsed with 0.004 mol/L phosphoric acid (acceptor phase), which was used for drop generation. This protocol was performed repeatedly before each extraction. The LLL-SDME procedure consists of a few steps. Initially, a urine sample with an organic solvent layer was stirred for 2 min. Next, the needle of the microsyringe was immersed in a chloroform layer and a 1 μL drop was generated. Then, the extraction of the analyte from the organic solvent to the acceptor drop took place for 15 min. After that, the enriched drop solution was aspirated back into the microsyringe. Depending on which procedure (method A or method B) was used, the following processing of the drop was different. Method A used the SDME technique during sample preparation, whereas method B utilized two combined preconcentration techniques, i.e., SDME during sample preparation together with on-capillary FASI. In the case of method A, the drop solution was transferred into a conical vial, diluted with 1 μL of deionized water, hydrodynamically introduced (50 mbar for 20 s) into the capillary, and analyzed in the CE system. In method B, the drop was transferred to vial and evaporated to dryness at 60 °C. Then, the residue was reconstituted in 10 μL of 0.0004 mol/L boric acid and introduced into the capillary in accordance with the following procedure: hydrodynamic injection of water plug (10 mbar for 10 s), electrokinetic injection of sample (5 kV for 30 s), and finally hydrodynamic injection of water plug (10 mbar for 5 s).

### 3.8. Calibration of the Method

To prepare the calibration standards for HTL determination in urine, 0.1 mol/L homocysteine thiolactone stock solution was diluted with deionized water as needed. Each HTL working standard solution was prepared according to following procedure: 1.667 mL of urine was transferred into a 5 mL calibrated flask and spiked with an increasing amount of HTL standard solution to receive concentrations of exogenous HTL from 0.22 to 0.50 μmol/L urine (method A), and from 0.10 to 0.5 μmol/L urine (method B). Then, in order to top the flask up to 5 mL, 0.2 mol/L phosphate buffer (pH 8.2) was added, the mixture was thoroughly mixed, and a stirrer was put into the flask. Next, 55 μL of chloroform was placed on the surface of the calibration solution. Subsequently, an off-line SDME procedure was performed according to method A or B, as described in Section 3.7. The CZE analysis of extracted working standard solutions was carried out under the conditions described in Section 3.4 (method A or B). The peak areas of HTL were plotted versus analyte concentration, and the calibration curve was fitted by least-square linear regression analysis.

## 4. Conclusions

This work describes a new, simple, precise and accurate CZE method for the determination of HTL in human urine, which, for the first time, utilizes a combination of two different preconcentration techniques. To the best of our knowledge, in this procedure, LLL-SDME has been realized outside the CE system for the first time, and on-line FASI was employed in order to improve the method’s sensitivity. Several methods for the determination of HTL in urine or plasma, which have a better or comparable sensitivity, have been developed before now [8,30,32]. However, in contrast to previously published assays, our procedure exhibits several benefits. The utilization of SDME has the following advantages: simple and inexpensive implementation in analytical procedure, significant reduction of toxic solvents, and the ability to purify samples and the analyte preconcentration. Using FASI as a sophisticated stacking procedure allows for further analyte preconcentration and sample matrix simplification. In contrast to other protocols, here, simple UV detection (known for its stability and low demand in terms of maintenance) was used for HTL identification. It is worth emphasizing that no time-consuming derivatization reaction was needed. On the other hand, the microextraction step is not a limitation in this case because a large set of samples can be prepared at the same time. The method is characterized by its acceptable precision, satisfactory accuracy and high sensitivity enhancement factor. A significant advantage of the described assay is its low reagent cost and usage, which makes our methodology more economical, as well as environmentally friendly. We believe that our new method can be suitable for quick and accurate HTL determination in clinical samples.

## Figures and Tables

**Table 1:** Validation data for method A.

Added (μmol/L)	Intra-Day			Inter-Day		
	Found ± SD (μmol/L)	Precision (%)	Accuracy (%)	Found ± SD (μmol/L)	Precision (%)	Accuracy (%)
0.24	0.24 ± 0.02	7.5	100.1	0.23 ± 0.02	8.1	96.1
0.35	0.32 ± 0.02	4.9	92.7	0.32 ± 0.02	5.6	92.6
0.47	0.46 ± 0.03	5.7	97.9	0.45 ± 0.03	6.4	96.0

n = 3.

**Table 2:** Validation data for method B.

Added (μmol/L)	Intra-Day			Inter-Day		
	Found ± SD (μmol/L)	Precision (%)	Accuracy (%)	Found ± SD (μmol/L)	Precision (%)	Accuracy (%)
0.15	0.15 ± 0.01	6.6	102.7	0.15 ± 0.01	8.3	98.2
0.30	0.28 ± 0.03	10.2	94.9	0.29 ± 0.03	9.5	97.6
0.45	0.46 ± 0.03	7.3	101.5	0.44 ± 0.03	6.4	102.7

n = 3.

**Table 3:** Concentration of homocysteine thiolactone in spiked human urine.

Sample Number	Added ^a^ (μmol/L)	Found ± SD (μmol/L)	RSD (%)
1 (m)	0.100	0.091 ± 0.002	2.3
2 (m)	0.100	0.110 ± 0.002	2.0
3 (m)	0.100	0.134 ± 0.011	8.2
4 (f)	0.100	0.102 ± 0.007	6.6
5 (f)	0.100	0.105 ± 0.006	6.0
6 (f)	0.100	0.112 ± 0.008	7.4
7 (f)	0.100	0.098 ± 0.001	1.3
8 (f)	0.100	0.115 ± 0.007	6.1
9 (f)	0.100	0.103 ± 0.006	5.7
10 (f)	0.100	0.161 ± 0.006	3.5
11 (f)	0.100	0.137 ± 0.004	2.8

^a^ n = 3; m—male; f—female.

## Data Availability

CE data are available from the authors.

## Supplementary Materials

The following are available online, Figure S1: The influence of BGE concentration on HTL signal parameter, Figure S2: Relationship between applied current and high applied voltage.

## Sample Availability

Urine samples are not available from the authors.
