# Supplementary material for: The Use of Single Drop Microextraction and Field Amplified Sample Injection for CZE Determination of Homocysteine Thiolactone in Urine
Source: Molecules. 2021 Sep 20;26(18):5687. doi: 10.3390/molecules26185687 (PMC8468900; doi:10.3390/molecules26185687)
Supplement: Supplementary file 1 [file molecules-26-05687-s001.zip › molecules-1342466-supplementary.pdf]

Suplementary materials

2 Results & discussion

2.1. Capillary zone electrophoresis

Figures:

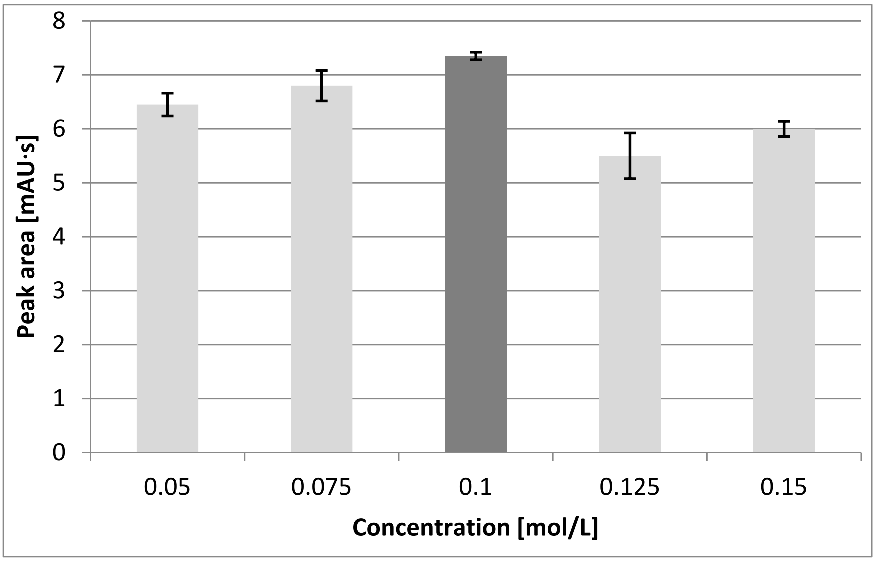

Figure S1. The influence of BGE concentration on HTL signal parameter.

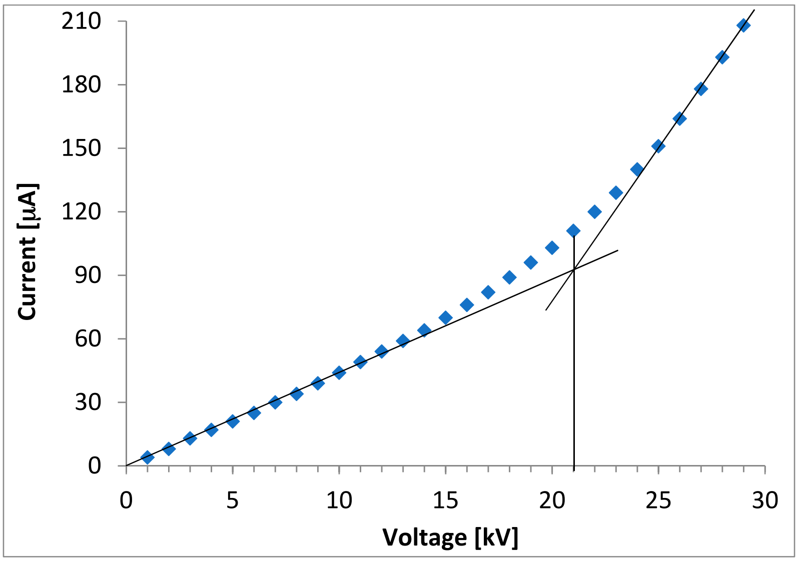

Figure S2. Relationship between applied current and applied high voltage.
